# The Overwhelming Majority but not All Motor Fibers of the Bifid Recurrent Laryngeal Nerve are Located in the Anterior Extralaryngeal Branch

**DOI:** 10.1007/s00268-015-3257-4

**Published:** 2015-10-05

**Authors:** Marcin Barczyński, Małgorzata Stopa, Aleksander Konturek, Wojciech Nowak

**Affiliations:** Department of Endocrine Surgery, Third Chair of General Surgery, Jagiellonian University Medical College, 37 Prądnicka Street, 31-202 Kraków, Poland; Third Chair and Department of General Surgery, Jagiellonian University Medical College, 37 Prądnicka Street, 31-202 Kraków, Poland

## Abstract

**Background:**

Few small studies reported that motor fibers are located exclusively in the anterior branch of the bifid recurrent laryngeal nerve (RLN). The aim of this study was to investigate the location of the motor fibers to the intrinsic muscles of the larynx among the bifid RLNs, and assess the prevalence of RLN injury with respect to nerve branching in a pragmatic trial.

**Methods:**

This was a prospective cohort study of 1250 patients who underwent total thyroidectomy with intraoperative neural monitoring. The primary outcome was the position of the motor fibers in the bifid nerves. Adduction of the vocal folds was detected by the endotracheal tube electromyography and abduction by finger palpation of muscle contraction in the posterior cricoarytenoid. The secondary outcomes were the prevalence of the RLN branching and the prevalence of RLN injury in bifid versus non-bifid nerves.

**Results:**

The bifid RLNs were identified in 613/2500 (24.5 %) nerves at risk, including 92 (7.4 %) patients with bilateral bifurcations. The motor fibers were present exclusively in the anterior branch in 605/613 (98.7 %) bifid nerves, and in both the RLN branches in 8/613 (1.3 %) bifid nerves. Prevalence of RLN injury was 5.2 versus 1.6 % for the bifid versus non-bifid nerves (*p* < 0.001), odds ratio 2.98 (95 % confidence interval 1.79–4.95; *p* < 0.001).

**Conclusions:**

The motor fibers of the RLN are located in the anterior extralaryngeal branch in the vast majority of but not in all patients. In rare cases, the motor fibers for adduction or abduction are located in the posterior branch of the RLN. As the bifid nerves are more prone to injury than non-branched nerves, meticulous dissection is recommended to assure preservation of all the branches of the RLN during thyroidectomy.

## Introduction

Recurrent laryngeal nerve (RLN) branching is a well-known phenomenon described in 18–85 % of patients [1–7]. In recent years, the clinical importance of RLN ramification has been underlined, as it was reported to be a risk factor for vocal folds dysfunction after thyroidectomy [8, 9]. Visual identification of the RLN and its anatomical variations requires a meticulous surgical dissection technique and can be facilitated by utilization of intraoperative neural monitoring (IONM) providing a new functional dynamics that allows for determination of neural function and the location of the RLN motor fibers in cases of RLN ramification [6, 10, 11]. Few explanatory trials identified the motor fibers for both adduction and abduction of the vocalis muscles to be located exclusively in the anterior branch of the bifid nerves [5, 7, 12]. However, this observation has not been confirmed to date in a large pragmatic trial. In addition, few case reports provided evidence that the RLN motor fibers may occur also in the posterior branch of the RLN [6, 13]. Hence, the aim of this study was to investigate prospectively the location of the motor fibers to the intrinsic muscles of the larynx among the bifid RLNs utilizing IONM, and assess the prevalence of RLN injury with respect to nerve branching in a large cohort of patients.

## Materials and methods

This was a prospective cohort study of 1250 patients (2500 RLNs at risk) who underwent total thyroidectomy with IONM and were treated in 2009–2013 at the Third Department of General Surgery, Jagiellonian University Medical College in Krakow, Poland.

The inclusion criterion was planned first-time total thyroidectomy with utilization of IONM. The exclusion criteria were the surgeon’s decision not to use IONM during the case, revision thyroidectomy, preoperative vocal fold palsy, staged thyroidectomy for loss of signal, incomplete clinical data, or follow-up information.

The primary outcome measure was the position of the motor fibers in the bifid nerves. The secondary outcome measures were the prevalence of RLN branching and the prevalence of RLN injury in the bifid versus non-bifid nerves.

The RLN and the presence of branching were routinely identified, evaluated using IONM, and preserved. Patients demographic and anatomy of the RLN were documented for each case and entered in a computerized prospective database.

Only the RLNs that bifurcated extralaryngeally, with both branches entering the larynx on the level of the lower margin of the inferior constrictor muscle, were counted as branched nerves.

Laryngoscopy was mandatory in all the patients before operation and afterward and was used to evaluate and follow RLN injury. All the patients provided written informed consent for the storage and use of their data. The protocol of this study was approved by the Institutional Review Board.

### Surgical technique

All the operations were performed under general anesthesia by four experienced endocrine surgeons (MB, AK, MS, and WN). The anesthesia protocol included intravenous midazolam premedication, induction with fentanyl, thiopental and suxamethonium, endotracheal intubation, and sevoflurane maintenance. No other muscle relaxants were used during the operations. A standard cervicotomy was used in all the patients. Visual identification of the RLN low in the neck (below the crossing with the inferior thyroid artery) was facilitated by the use of the IONM system with the employment of the nerve mapping technique. Once the nerve was visually identified, repeated stimulations with the monopolar probe of the IONM system served to trace the nerve path in the operative field and test its functional integrity during dissection. In each patient, the RLN was exposed and the branches of the superior and inferior thyroid arteries were divided close to the thyroid capsule (peripheral ligation). The characteristics of the patients are presented in Table 1.

**Table 1:** Characteristics of patients

	Patients in the study (*n* = 1250)
RLNs at risk, no	2500
Female:male ratio, no	1130:120
Mean age ± SD	54.6 ± 13.5
Preoperative diagnosis, no (%)	
Non-toxic multinodular goiter	838 (67.0)
Toxic multinodular goiter	130 (10.4)
Graves’ disease	24 (1.9)
Suspected follicular neoplasm	121 (9.7)
Differentiated thyroid cancer	134 (10.7)
Medullary thyroid cancer	3 (0.2)
Extent of surgery, no (%)	
Total thyroidecotmy	1232 (98.6)
Central lymph nodes clearance	258 (20.6)
Lateral lymph nodes clearance	28 (2.2)
Staged thyroidectomy for LOS	18 (1.4)

Data are numbers with percentages in parentheses unless otherwise indicated

*RLN* recurrent laryngeal nerve, *LOS* loss of signal

### IONM technique

During this study period, the NIM^®^ 2.0 followed by the NIM^®^ 3.0 system (Medtronic, Jacksonville, US) was in use between 2009 and 2013. The NIM^®^ system operated with surface electrodes integrated with an endotracheal tube 7.0–7.5 in diameter, which was inserted by an anesthetist between the vocal folds under direct vision during intubation. The standardized technique of neuromonitoring of the RLNs was used, including indirect vagal response evaluation at the beginning and at the end of the operation (IONM = L1 + V1 + R1 + R2 + V2 + L2) according to the recommendations formulated recently by the International Intraoperative Neural Monitoring Study Group [10]. The nerves were stimulated using a monopolar electrode and the interrupted stimulation technique at 1 mA, 100 ms impulse duration, and 4 Hz frequency. In case of the bifurcated RLN nerves, the assessment included post-stimulation response of each nerve branch. Adduction of the vocal folds was detected by the endotracheal tube electromyography and abduction by finger palpation of muscle contraction in the posterior cricoarytenoid (“laryngeal twitch”) [10, 11].

### Perioperative management and follow-up

Laryngoscopy (either indirect or direct videolaryngoscopy) by a throat specialist was mandatory before surgery and on day 1 after surgery. In patients with RLN paresis, an additional examination was scheduled at 1, 2, 4, 6, and 12 months after surgery, or until the vocal cord function was recovered. Vocal cord paresis for more than 12 months after the operation was regarded as permanent palsy.

### Statistical analysis

The incidence of nerve events was calculated based on the number of nerves at risk. Data are presented as numbers with corresponding percentage, or mean with standard deviation, or 95 % confidence interval (CI), unless stated otherwise. The statistical significance of categorical variables was evaluated by the *χ*^2^ test, whereas the Students *t* test was used for the analysis of continuous variables. Odds ratios (ORs) with 95 % CI were calculated for categorical variables. The logistic regression analysis was used to identify predictors of the risk of RLN injury. The variables considered for the univariable analysis were gender, age, toxic goiter, thyroid cancer, retrosternal goiter, side of the neck, and extralaryngeal branching. All the data were collected prospectively, stored in a computerized database, and analyzed by a statistician, assuming that *p* < 0.05 was indicative of significance. Statistical analyses were performed with MedCalc (version 13, MedCalc Software, Belgium).

## Results

Of 4158 patients referred for thyroid surgery during the study interval, 3783 were planned for bilateral thyroid surgery and were potential candidates for the study. Twenty-two hundred and forty-nine patients did not meet the inclusion criteria, as there was no plan for utilization of IONM during surgery, while 284 subjects were excluded (revision thyroidectomy—224, refusal to participate—48, loss of signal leading to staged procedure—12), leaving 1250 patients (2500 nerves at risk) who were finally included in this study. There were 1130 women and 120 men, with a mean age at diagnosis of 54.6 ± 13.5 years. The clinical characteristics of the patients analyzed in this study are shown in Table 1.

### Primary outcome measures analysis

The motor fibers were present exclusively in the anterior branch of the RLN in 605/613 (98.7 %) bifid nerves, and in both branches of the RLN in 8/613 (1.3 %) bifid nerves. The median distance from the ramification level to the entry point into the larynx was 19 mm (95 % CI 17–21 mm) for the nerves with the motor fibers exclusively in the anterior branch versus 9 mm (95 % CI 7–11 mm) for the nerves with the motor component in both branches (*p* = 0.001). Of eight bifid nerves with the posterior motor branch, a representative glottis EMG response waveforms together with a palpable laryngeal twitch were obtained in four cases following the stimulation of the posterior branch, whereas in the remaining four cases, no glottis EMG response waveform, but a clearly visible and palpable laryngeal twitch was noticed. The EMG amplitude following stimulation of the anterior RLN branch with 1 mA current was significantly lower among 605 nerves with the motor component exclusively in the anterior branch than among eight nerves with the motor component in both branches (948 ± 199 vs. 1534 ± 150 µV, respectively; *p* = 0.001, latency 1.3 ms for both). Details are shown in Table 2.

**Table 2:** Primary and secondary outcomes of this study

Single trunk RLN, no (%) Bifid RLN left Bifid RLN right Bifid RLN bilateral	1887 (75.5) 290 (23.2) 323 (25.8) 92 (7.4)
Bifid RLN, no (%) Posterior sensory branch (no EMG, no laryngeal twitch) Posterior motor branch (positive EMG and laryngeal twitch) Posterior motor branch (no EMG but palpable laryngeal twitch)	613 (24.5) 605 (98.7) 4 (0.6) 4 (0.6)
Distance from RLN ramification point into the larynx entry, mm (95 % CI)	
Posterior sensory branch	19 (17–21)
Posterior motor branch	9 (7–11)
RLN EMG amplitude following 1 mA stimulation of the anterior branch^a^, mean ± SD	
Posterior sensory branch (no EMG, no laryngeal twitch)	948 ± 199 µV^†^
Posterior motor branch (positive EMG and laryngeal twitch)	785 ± 158 µV
Posterior motor branch (no EMG but palpable laryngeal twitch)	1534 ± 150 µV^†^
RLN EMG amplitude following 1 mA stimulation of the posterior branch, mean ± SD	
Posterior motor branch (positive EMG and laryngeal twitch)	474 ± 76 µV
Posterior motor branch (no EMG but palpable laryngeal twitch)	No EMG
Early RLN injury, no (%) Single trunk RLN Bifid RLN	62 (2.5) 30 (1.6)^‡^ 32 (5.2)^‡^
Permanent RLN injury, no (%) Single trunk RLN Bifid RLN	10 (0.4) 3 (0.2) 7 (1.1)

*RLN* recurrent laryngeal nerve, *EMG* electromyography

^†^ *p* = 0.001

^‡^ *p* < 0.001

^a^Response from the anterior branch following stimulation with 1.0 mA

### Secondary outcome measures analysis

The non-RLN was identified in 9 (0.7 %) patients on the right side of the neck. The bifid RLNs were identified in 613/2500 (24.5 %) nerves at risk, including 92 (7.4 %) patients with bilateral bifurcations. Prevalence of early RLN injury was 5.2 versus 1.6 % for the bifid versus non-bifid nerves (*p* < 0.001), OR 2.98 (95 % CI 1.79–4.95; *p* < 0.001). Detailed data of the univariable analysis of the variables associated with early and permanent RLN injury are shown in Table 3.

**Table 3:** Univariable analysis of variables associated with early and permanent RLN injury

Characteristics	No. of patients	Early RLN injury (*n* = 62)		Permanent RLN injury (*n* = 10)	
		Odds ratio (95 % CI)	*P*	Odds ratio (95 % CI)	*P*
Gender					
Male	240	0.64 (0.23–1.78)	0.397	2.36 (0.49–11.20)	0.277
Female	2260	1.00		1.00	
Age (years)					
≥65	800	1.01 (0.58–1.74)	0.964	1.41 (0.39–5.04)	0.588
<65	1700	1.00		1.00	
Surgery for toxic goiter					
Yes	308	1.22 (0.59–2.52)	0.590	3.07 (0.78–11.93)	0.105
No	2192	1.00		1.00	
Surgery for suspected thyroid cancer					
Yes	516	1.24 (0.68–2.25)	0.479	1.65 (0.42–6.40)	0.468
No	1984	1.00		1.00	
Surgery for retrosternal goiter					
Yes	377	2.18 (1.23–3.85)	0.007	1.40 (0.29–6.63)	0.669
No	2123	1.00		1.00	
Side of the neck					
Right	1250	1.84 (1.09–3.12)	0.022	1.50 (0.42–5.33)	0.529
Left	1250	1.00		1.00	
Bifid RLN					
Yes	613	2.98 (1.79–4.95)	<0.001	1.32 (0.34–5.12)	0.687
No	1887	1.00		1.00	

*RLN* recurrent laryngeal nerve, *95* *% CI* 95 % confidence interval

## Discussion

IONM during thyroid surgery has gained widespread acceptance as an adjunct to the gold standard of visual nerve identification, adding a new functional dynamics during thyroid surgery [10]. The International Neural Monitoring Study Group has defined three discrete modes of IONM application in thyroid surgery: identification (neural mapping) of the RLN, aid in nerve dissection, and prognostication of postoperative neural function and lesion site identification [10, 14]. In addition, IONM can be also used to facilitate identification of the external branch of the superior laryngeal nerve during thyroid surgery, contributing to voice preservation [15, 16].

Bifurcation of the RLN is a common anatomical variation encountered in thyroid surgery and when present is the source of increased surgical morbidity [6, 8, 9]. The values of the RLN reported in the literature range widely from 18 to 85 % [1–7]. However, in most studies, the RLN branching rate does not exceed 20 %, which suggests underestimation due to difficulties in visual identification. Barczyński et al. showed in a randomized study with 2000 nerves at risk that IONM helped to identify 33 % more RLN bifurcations than visualization alone [4]. Sancho et al. reported extralaryngeal branching in 37.4 % of 302 nerves at risk and found that branched nerves were twofold more likely to be associated with vocal fold dysfunction postoperatively; OR 2.2 (95 % CI 1.1–4.5) [8]. Similarly, the comparative analysis of postoperative outcomes between 36 of 195 (18.5 %) branched and the remaining non-branched RLNs made by Casella et al. revealed that this anatomical variation was more frequently associated both with unilateral permanent RLN palsy (RR 13.25; 95 % CI 1.42–123.73; *p* = 0.020) and unilateral transient RLN palsy (RR 7.36; 95 % CI 1.84–29.4; *p* = 0.006) [9]. Those findings were confirmed by the outcomes of the present study, as the prevalence of early RLN injury was almost three-fold higher in the branched versus non-branched nerves: 5.2 versus 1.6 %, respectively (*p* < 0.001), OR 2.98 (95 % CI 1.79–4.95). The rationale supporting easier vulnerability of the branched nerves include difficulties in visual identification and preservation of the small caliber fibers [6]. Female patients were reported to be more likely to have a greater length of the bifid RLNs and thus could be exposed to a greater risk of nerve injury [6]. The use of IONM during thyroid surgery is advised by the International Neural Monitoring Study Group, as it can be of additional aid in nerve dissection and providing functional information which is not available through visual inspection alone [10].

Few prior reports on a limited number of patients suggested that the motor nerve fibers are located exclusively in the anterior branch of the bifid RLNs [5, 7, 12]. Serpell et al. used nerve integrity monitoring to assess 176 RLNs in 118 patients. Of these 41 (23.3 %) were bifid RLNs. In all 41 (100 %) cases of bifid RLNs, motor fibers for both adduction and abduction of the vocal cords were located exclusively in the anterior branches of RLN, and none in the posterior branches [5]. Kandil et al. studied a total of 310 RLNs in 220 patients and found that 133 RLNs (42.9 %) bifurcated before entering the larynx. In all bifurcated RLNs in this study, the motor fibers were located exclusively in the anterior branches [7]. However, Fontenot et al. recently reported a cohort of 719 nerves at risk, of which 264 (36.7 %) were bifid; on electrophysiological testing, the above authors found the motor fibers in all the anterior branches and 3 (1.1 %) posterior extralaryngeal RLN branches [6]. In all of these three cases, stimulation of the anterior branches also elicited a motor response, while stimulation of the posterior branches produced an evoked glottis EMG response waveform with the mean amplitude of 634 µV and the mean latency of 1.2 ms [6]. In the current prospective study with 2500 RLNs at risk, which is the largest to date, we found that 613 (24.5 %) nerves were bifid, and IONM stimulation identified the motor fibers in all the anterior branches and 8 (1.3 %) posterior extralaryngeal RLN branches. However, contrary to the report of Fotenot et al. only four of eight posterior branches of the bifid nerves with a palpable laryngeal twitch following the stimulation showed a glottis EMG response waveform on electrophysiological testing with the mean amplitude of 474 ± 76 µV and the mean latency of 1.2 ms, while the remaining four posterior branches were silent on EMG, but showed a clearly visible and palpable laryngeal twitch following the stimulation. In addition, the mean EMG amplitude following 1 mA stimulation of the anterior branches was different in 605 cases with the posterior sensory branch (no EMG response, no laryngeal twitch) when compared to four cases with the posterior motor branch (no EMG response, but clearly visible and palpable laryngeal twitch): 948 ± 199 versus 1534 ± 150 µV, respectively (*p* < 0.001), but not when compared to the remaining four cases with the posterior motor branch (positive EMG and laryngeal twitch): 948 ± 199 vs. 785 ± 158 µV (*p* = 0.927). Interpretation of these outcomes can be done in the context of the vocalis muscles anatomy and physiology. Adduction of the vocal fold is a result of a combined action of three muscles: the thyroarytenoid (TA), lateral cricoarytenoid (LCA), and interarytenoid muscles (IT), whereas abduction of the vocal fold is dependent on the action of the only one posterior cricoarytenoid (PCA) muscle [17, 18]. In the vast majority of extralaryngeal bifurcations of the RLN, the anterior branch of the bifid nerves carries the motor fibers to both the adductors and abductor of the vocal fold and electrical stimulation of the anterior branch results in vocal fold adduction (as a combined action of the TA, LCA, and IT muscles is much stronger than the activity of the only one abductor muscle—PCA) which is recorded by surface electrodes on the endotracheal tube. However, in rare cases of the posterior motor branch of the bifid RLNs, an electrophysiological response can be recorded in some but not all cases. A positive EMG response can be expected as a result of the vocal fold adduction, meaning that the posterior branch must contain fibers innervating at least one of the adductors: the TA, LCA, or IT muscle. However, in cases with no EMG response following the stimulation of the posterior RLN branch but with a clearly visible and palpable laryngeal twitch, the posterior branch can contain the motor fibers innervating the abductor of the vocal fold—the PCA muscle, the contraction of which does not result in an EMG response waveform on the IONM monitor when using standard superficial electrode on the endotracheal tube. Such an explanation is supported by the data of the current study, as the mean EMG amplitude following 1 mA stimulation of the anterior branches was significantly lower in 605 cases with the posterior sensory branch (no EMG response, no laryngeal twitch) when compared to four cases with the posterior motor branch (no EMG response, but a clearly visible and palpable laryngeal twitch): 948 ± 199 versus 1534 ± 150 µV, respectively (*p* < 0.001). The EMG response waveform amplitude in the latter group of the bifid nerves could be higher as a result of a combined action of all the adductors localized in the anterior motor branch of the bifid RLN without diminishing the effect on the amplitude of the PCA muscle abducting contraction, which is the case in most instances when the posterior branch is solely sensory. It is of interest to note that more recent studies have described no consistent functional pattern of branching of the anterior and posterior laryngeal branches. Maranillo et al. examined 75 human larynges obtained from necropsies using a careful dissection technique with intraoperative microscopy [19]. The results of this study did not support the existence of an abductor and adductor division of the RLN. In 88 % of cases, the nerve supply of one of the adductor muscles (arytenoid muscle) and the abductor muscle (PCA muscle) arose from a common trunk [19]. However, the PCA muscle nerve supply in all cases (100 %) came from the anterior division of the RLN [20]. Similar observations were made by Eller et al. who examined 43 human cadaver larynges using microscopic dissection technique [21]. All of the PCA muscles received innervation from the anterior division of the RLN. The number of direct branches from the RLN ranged from 1 to 5 (average 2.3). More than 70 % of PCA muscles also received 1-3 branches off of the branch to the IA muscle. Less than half of PCA muscles received any kind of nerve branches from the posterior division of the RLN. Branches to the PCA most commonly departed the main RLN in its vertical segment and all entered the muscle from its deep surface [21].

Another important point noticed in this cohort of patients was that where the motor fibers were exclusively in the anterior branch, the branching occurred at 19 mm (95 % CI 17–21 mm) whereas in the 8 cases only, where the motor fibers were in the posterior branch, the branching occurred at 9 mm (95 % CI 7–11 mm) only (*p* = 0.001). This observation needs to be validated by other researchers. It is well-known that most true extralaryngeal RLN branches (i.e., major branches destined to enter the larynx) arise from the distal RLN segment, and when present, usually exist at the level of the ligament of Berry, and are usually not present below the inferior thyroid artery [4–9]. The distal-most RLN intralaryngeal branches are always given off by the time the RLN is above the cricothyroid joint [22]. From this point of view, extralaryngeal RLN branches can be assumed to be analogous to intralaryngeal branches, except that they are premature, occurring more proximally below the lower edge of the inferior constrictor [22]. For that reason, many surgeons consider extralaryngeal branching occurring at a distance less than 10 mm from entering into the larynx as the anatomical variant of the RLN, which is quite common [4, 5]. However, this anatomical variant of the nerve is particularly prone to injury due to an increased anatomic complexity and a narrow diameter of the branched RLN [8, 9].

Despite a prospective design, the current study has several limitations. Not all eligible patients were included, as the employment of IONM in Poland is not reimbursed by the Polish National Health Fund which is the main reason for using this technique on select patients depending on the individual surgeon’s preferences and availability of the equipment. The operations were performed not by one, but four (MB, MS, AK, and WN) surgeons. However, in order to minimize the risk of any bias, all the surgeons involved in this study had a comparable background experience both in thyroid surgery and utilization of IONM during thyroidectomy. In addition, it was a single-center study, while a multi-institutional study design would be more powerful to elucidate whether patients with RLN branching are at higher risk of permanent RLN palsy, which has not been proven.

In conclusion, the motor fibers of the RLN for both adduction and abduction are located in the anterior extralaryngeal branch in the vast majority of but not in all patients. In rare cases, the motor fibers for adduction or abduction are located in the posterior branch of the RLN. IONM is able to help by confirming the location of the motor component of the RLN. As the bifid nerves are more prone to at least a temporary injury than non-branched nerves, meticulous dissection is recommended to assure preservation of all branches of the RLN during thyroidectomy.
